# The Usher syndrome 1G protein SANS participates in the transport of ciliary cargo in photoreceptor cells

**DOI:** 10.1186/2046-2530-1-S1-P48

**Published:** 2012-11-16

**Authors:** N Sorusch, K Bauß, N Overlack, A Kunz, E van Wijk, T Maerker, R Roepman, H Kremer, U Wolfrum

**Affiliations:** 1 Cell and Matrix Biology, Inst. of Zoology, Johannes Gutenberg University of Mainz, Germany; 2 Dept of Human Genetics, Nijmegen Centre for Molecular Life Sciences, Nijmegen, the Netherlands; 3 Institute for Genetic and Metabolic Disease Nijmegen Centre for Molecular Life Sciences, Nijmegen, the Netherlands; 4 Dept of Otorhinolaryngology, Head and Neck Surgery, Nijmegen Centre for Molecular Life Sciences, Nijmegen, the Netherlands; 5 Nijmegen Centre for Molecular Life Sciences, Nijmegen, the Netherlands; 6 Donders Institute for Brain, Cognition and Behaviour, Radboud University Nijmegen Medical Centre, Nijmegen, the Netherlands

## 

Human Usher syndrome (USH) is the most common form of combined deaf-blindness, characterized by profound congenital deafness, constant vestibular dysfunction and pre-pubertal onset of retinitis pigmentosa. The USH1G protein SANS (scaffold protein containing ankyrin repeats and SAM domain) is associated with microtubules and mediates a transport related periciliary protein network in photoreceptor cells. Here we aim to enlighten the involvement of SANS in ciliary transport of photoreceptor cells by identifying proteins associated with SANS in transport complexes. In Y2H screen of retinal cDNA library we identified the direct binding of SANS to dynactin-1 (p150Glued), a subunit of the dynactin complex and cargo linker to the cytoplasmic dynein motor. This interaction was validated in complementary interaction assays in vitro and in cell culture. In addition, we demonstrated the integration of SANS into the cytoplasmic dynein transport complex by GST-pull down of SANS with cytoplasmic dynein intermediate chain (cDIC74). Correlative immunofluorescence and -electron microscopy analyses of photoreceptor inner segments revealed co-localization of essential dynein-dynactin components and SANS at microtubules, the known transport routes for opsin bearing vesicles. Co-immunoprecipitation revealed that SANS is part of an opsin containing protein complex together with transport regulating rab-GTPases. Finally, the analysis of SANS deficient mice revealed altered distribution of opsin in the inner segment, indicating a transport delay. In conclusion we identified SANS as a component of the cytoplasmic dynein motor complex which is crucial for opsin transport to photoreceptor connecting cilia. Defects in these transport mechanisms lead to retinal degeneration as characteristic for USH1G patients.

